# Mercury in Retrograde: The Tale of Toxicity

**DOI:** 10.1155/crin/1913507

**Published:** 2026-05-27

**Authors:** Sandra Abadir, Rafi Orphali, Garo Kalfayan, Arian Gower, Mercury Lin, Jasprit Takher

**Affiliations:** ^1^ Department of Internal Medicine, Los Robles Regional Medical Center, HCA Healthcare, Thousand Oaks, California, USA, hcahealthcare.com; ^2^ Department of Pathology, Cedars-Sinai Medical Center, Los Angeles, California, USA, cedars-sinai.edu

## Abstract

**Introduction:**

Mercuric chloride is a highly toxic inorganic compound with historical medical use but severe potential for harm when ingested. Acute mercury poisoning is rare but can lead to multiorgan toxicity, particularly affecting the kidneys and gastrointestinal tract. Prompt recognition and intervention are essential to prevent long‐term sequelae and mortality.

**Case Presentation:**

An 18‐year‐old male with a psychiatric history presented one hour after intentional ingestion of five mercuric chloride tablets. He exhibited mild abdominal symptoms, and initial laboratory studies revealed proteinuria, glycosuria, and rising creatinine. Imaging showed echogenic kidneys, and serum mercury levels were markedly elevated at 840 μg/L. He was treated with oral dimercaptosuccinic acid (DMSA), intravenous fluids, and gastrointestinal decontamination. Despite therapy, his renal function declined, with creatinine peaking at 9.6 mg/dL, requiring hemodialysis. Renal biopsy demonstrated acute tubular necrosis without immune complex deposition. After six sessions of hemodialysis and 16 days of chelation therapy, renal function and mercury levels improved, allowing discharge to psychiatric care.

**Discussion:**

Mercuric chloride exerts its nephrotoxic effects primarily through oxidative injury to proximal tubular cells, resulting in acute tubular necrosis. While chronic mercury exposure is more often associated with glomerular diseases, acute ingestion more typically causes tubular injury. Chelation with DMSA facilitates mercury elimination but may be insufficient when renal impairment occurs. In such cases, hemodialysis becomes essential for toxin clearance and metabolic support. This case underscores the importance of early, combined therapeutic interventions.

**Conclusion:**

Acute mercuric chloride ingestion is a medical emergency that can cause life‐threatening renal toxicity. Timely initiation of chelation therapy and supportive measures, including hemodialysis, are crucial for recovery. This case demonstrates that even in severe presentations, multidisciplinary management can result in favorable outcomes.

## 1. Introduction

Mercury is a heavy metal that exists as a liquid at standard temperature and pressure, but it can also exist in gaseous and solid states. It appears in three distinct forms: elemental, organic, and inorganic, all of which are toxic and can lead to significant health complications. Mercuric chloride, an inorganic form of mercury, is particularly dangerous and can cause harm through ingestion, inhalation, or skin absorption [1]. Chronic exposure to significant doses of mercury vapor is the most common cause of mercury poisoning, leading to symptoms such as gastrointestinal distress, fatigue, pneumonitis, weight loss, weakness, and neurological manifestations, including tremors [2]. In contrast, acute mercury poisoning is far less common and typically results from inhalation or dermal absorption of mercury‐containing substances [3–5]. Although rare, acute poisoning can also occur following direct ingestion of mercury [6].

Historically, mercuric chloride (or calomel) was used as a diuretic and antiseptic, especially in the treatment of syphilis due to its antimicrobial properties. However, these treatments often resulted in significant side effects and morbidity due to renal failure [7, 8]. The toxic effects of mercuric chloride stem from its ability to disrupt cellular processes and generate reactive oxygen species, leading to organ damage, particularly in the kidneys [9]. Additionally, its corrosive nature can cause gastrointestinal injuries. Although mercuric chloride is typically excreted through urine or feces and does not easily cross the blood–brain barrier, it has been detected in cerebrospinal fluid in a case involving a potentially lethal 5‐g ingestion [10].

The standard treatment for heavy metal toxicity is chelation therapy. For inorganic mercury toxicity, potential chelators include dimercaprol, penicillamine, or unithiol, but dimercaptosuccinic acid (DMSA) is the preferred agent. In severe cases of mercuric chloride toxicity, hemodialysis may also be employed to facilitate the removal of mercury from the bloodstream [11]. While many patients with syphilis who were treated with mercury suffered from severe kidney failure and death [12], this case report describes a patient who ingested mercuric chloride and developed acute toxicity. The patient was successfully treated with a combination of DMSA chelation therapy and hemodialysis, highlighting an effective approach to managing acute mercury poisoning.

## 2. Case Presentation

An 18‐year‐old man with a history of anxiety and depression, treated with ketamine infusions, was brought to the emergency department by police following a suicide attempt one hour before presentation. He had ingested five tablets of unknown dose of mercury dichloride obtained from an antique store. Upon arrival, he reported mild upper abdominal pain, nausea, and nonbilious, nonbloody vomiting. On examination, the patient was found to be tachycardic (107 beats/min), afebrile, and hypertensive (150/80 mm Hg), with an oxygen saturation of 98% on room air. Physical examination revealed no abnormal findings.

Laboratory tests results revealed a hemoglobin of 18.6 g/dL, total bilirubin of 2.6 mg/dL, direct bilirubin of 0.3 mg/dL, and aspartate aminotransferase (AST) at 40 U/L. Urine toxicology was positive for tetrahydrocannabinol, and urinalysis showed a pH of 9, protein > 500 mg/dL, glucose > 500 mg/dL, trace ketones, 1+ blood, and 6–10 white blood cells (WBC) per high power field (HPF). An abdominal X‐ray showed a nonobstructive gas pattern, negative for a radiopaque foreign body, while an abdominal ultrasound indicated increased echogenicity of the bilateral kidneys.

The patient was admitted and treated with oral DMSA 800 mg every 8 hours, oral charcoal/sorbitol 50 g once, and intravenous fluids as recommended by poison control. DMSA was given with food to limit nausea and GI upset. Our patient also required IV ondansetron for nausea. He was placed on continuous telemetry and suicide precautions. Psychiatry was consulted, and while a 5150 hold was initially considered, the patient expressed that he had acted impulsively and denied any further suicidal thoughts. As a result, the hold was not required. Although psychiatric medications were not started right away, the patient was cooperative and agreed to stay in the hospital until he was medically stabilized.

Gastroenterology was consulted, and polyethylene glycol was administered every 2 hours for a total of 6 doses to help eliminate gastric content and minimize mercury absorption. Within a day, the WBC count increased to 27.57 × 10^9^/L, creatinine rose to 2.50 mg/dL, and serum mercury levels were elevated at 840 μg/L. Since serum mercury is better to assess for recent and acute exposure to mercury, it was used instead of a 24‐h urine sample.

**FIGURE 1:**
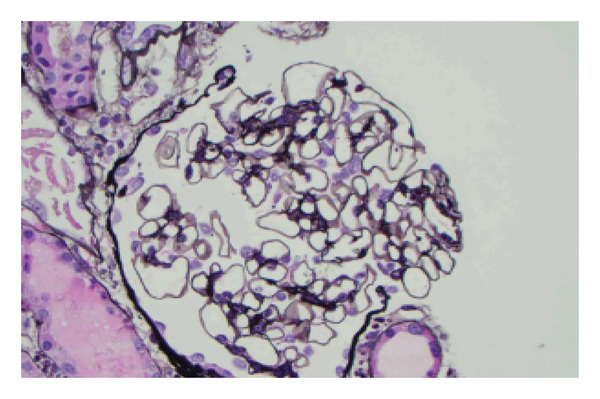
Description: Unremarkable glomerulus devoid of pathological alteration. (Jones’ silver stain, original magnification X400).

Given that mercury nephrotoxicity can manifest as either dose‐dependent acute tubular necrosis (ACN) or immune‐mediated glomerular disease, with the latter potentially requiring glucocorticoids or immunosuppression in addition to chelation, a renal biopsy, after consent, was performed to guide management. Tissue samples were obtained for light microscopy, immunofluorescence, and electron microscopy analysis. The specimen included the renal capsule and cortex. A total of 22 glomeruli were sampled, with one showing global sclerosis. The remaining glomeruli appeared patent and normocellular, without evidence of necrotizing or crescentic injury (Figure 1).

**FIGURE 2:**
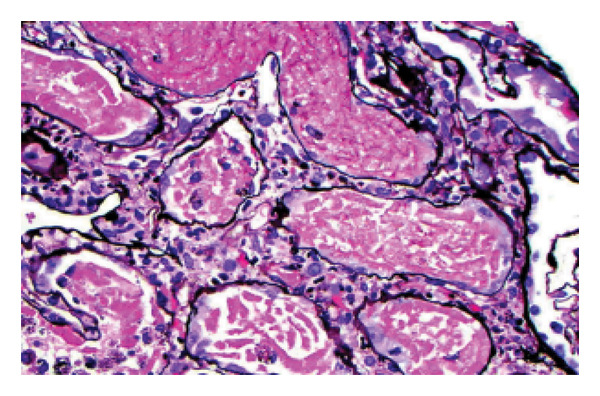
Description: Severe acute tubular necrosis involving proximal tubular segments with tubular epithelial cell necrosis, loss of brush borders, intraluminal shedding of cells, and focal mitotic activity. There are also interstitial edema and mild interstitial inflammation. (Jones’ silver stain, original magnification X400).

**FIGURE 3:**
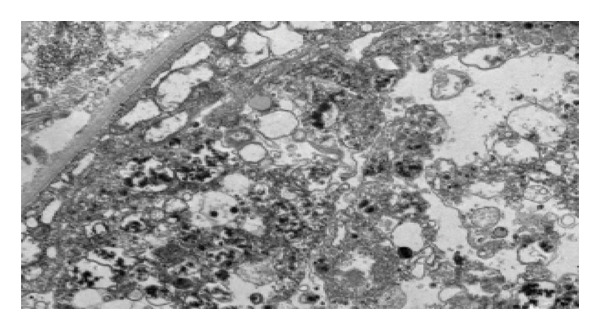
Description: Membrane bound vesicles with electron dense material in severely injured tubular epithelial cells (electron microscopy, original magnification x5000).

Prominent tubular necrosis was observed, particularly in the proximal tubular segments (Figure 2). There were no oxalate crystals within the tubular lumina nor were there any hemoglobin or myoglobin casts. Additionally, there was patchy interstitial inflammation, which consisted of a mixed population of small mononuclear cells, eosinophils, and neutrophils. Mild renal cortical scarring was noted, but there was no evidence of vasculitis or vascular thrombosis. Immunofluorescence and electron microscopy studies showed no immune complex deposition. Electron microscopy revealed membrane‐bound vesicles containing electron‐dense material in the injured proximal tubular epithelial cells (Figure 3).

Based on these findings, the histopathologic diagnoses were as follows:

1. Severe ACN, consistent with acute nephropathy due to mercuric chloride poisoning.
2. Mild acute interstitial nephritis.

After another psychiatry consultation, he was started on quetiapine 25 mg and ziprasidone 10 mg every 6 h as needed for agitation. By Day 3 of hospital stay, his urine output dropped significantly, and his creatinine had risen to 9.60 mg/dL, necessitating hemodialysis. An endoscopy revealed 2 ulcerations on the greater curvature, with biopsy results showing moderate to severe acute on chronic gastritis with erosion and ulceration. Pantoprazole and sucralfate were added to his treatment regimen. Initially, he was set for outpatient hemodialysis; however, his creatinine decreased to 1.80 mg/dL, and serum mercury levels dropped to 435.5 μg/L. DMSA was discontinued after 16 days, and he received 6 3‐hour sessions of hemodialysis. He was eventually transferred to an inpatient psychiatric facility in a hemodynamically stable condition for ongoing care and support.

## 3. Discussion

Acute exposure to mercuric chloride is a serious and potentially fatal condition, primarily due to its gastrointestinal and nephrotoxic effects. One of the most severe manifestations of toxicity is ATN, which results from the compound’s direct toxic impact on the kidneys. The mechanism of injury involves the binding of mercuric ions to sulfhydryl groups in proteins, leading to oxidative stress and cellular damage within the proximal tubular cells of the kidney [13, 14]. This damage disrupts normal renal function, leading to inflammation, tubular dysfunction, and, ultimately, ATN. In this case, the patient’s acute exposure to mercuric chloride caused significant renal impairment, highlighting the severe consequences of this toxin on kidney function. Although mercury poisoning (specifically in long‐term exposure) has been reported to be associated with membranous nephropathy, minimal change disease, and mesangial proliferative glomerulonephritis [15, 16], there is no evidence of glomerular disease in our patient who experienced acute mercury poisoning.

DMSA, a chelating agent commonly used in the treatment of heavy metal poisoning, has been shown to be effective in managing acute mercuric chloride toxicity. DMSA binds to mercury ions via its free sulfhydryl groups, facilitating the renal excretion of the toxin and thereby reducing systemic mercury levels [17]. The timing of intervention is crucial, as early treatment, ideally within minutes to a few hours of exposure, and maximizes the protective effects of chelation. Delays in treatment, however, can diminish its efficacy as the mercury burden in the body increases [18, 19].

Alternative chelating agents have also been described in the management of mercury poisoning, each with distinct pharmacologic properties, routes of administration, and safety profiles. A comparison of commonly used agents is summarized in Table 1.

**TABLE 1:** Comparison of chelating agents for mercury poisoning.

Agent	Route	Advantages	Disadvantages
DMSA (Succimer)	Oral	Lowest toxicity profile; FDA‐approved; oral administration; does not redistribute mercury to brain; effective for inorganic and organic mercury	Limited intracellular penetration; hydrophilic so weak access to intracellular deposits
DMPS (Unithiol)	Oral or IV	Highly effective; dramatically reduces mercury half‐life (from 33 to 11 days); can be given IV in acute settings; does not redistribute mercury to brain	Not FDA‐approved in United States; more adverse effects than DMSA (nausea 50%, pruritus 25%); higher toxicity than DMSA
Dimercaprol (BAL)	IM only	Penetrates intracellular compartments; can mobilize intracerebral deposits; historically proven efficacy	Significant toxicity; painful IM injections; can redistribute mercury to brain; promotes intestinal reabsorption of biliary mercury (27% reabsorbed); narrow therapeutic index
D‐Penicillamine	Oral	Oral administration; reduces methylmercury half‐life to ∼26 days	Least effective of the dithiol chelators; no protection against inorganic mercury neurotoxicity in vitro; may potentiate toxicity of some mercury forms; significant adverse effect profile

*Note:* DMSA: meso‐2,3‐dimercaptosuccinic acid. IV: intravenous. DMPS: dimercaptopropane‐1‐sulfonic acid. IM: intramuscular.

Both DMSA and 2,3‐dimercapto‐1‐propane sulfonate (DMPS) are hydrophilic dithiol chelators that enhance renal mercury excretion, in part via multidrug resistance–associated protein 2 (MRP2) transporters [20]. Comparative data suggest that DMPS may achieve more rapid mercury clearance; during the Iraq methylmercury outbreak, DMPS reduced the blood mercury half‐life to approximately 10 days, compared to 26 days with D‐penicillamine [21]. However, DMSA remains the preferred first‐line agent in many settings due to its favorable safety profile and regulatory approval [22].

Dimercaprol (BAL), an older chelating agent, is capable of mobilizing intracellular mercury but is limited by significant toxicity. Notably, animal studies have demonstrated that BAL may increase central nervous system mercury concentrations, raising concern for redistribution [23].

D‐penicillamine is less effective for mercury chelation compared to DMSA and DMPS and is more commonly used in the management of copper overload, such as in Wilson’s disease [24]. In vitro studies have shown that D‐penicillamine does not confer neuroprotection and may potentiate the toxicity of certain mercury compounds [25].

In a recent systematic review of 126 mercury poisoning cases, chelating agents were administered to 84.1% of patients, with DMSA, BAL, DMPS, and D‐penicillamine being the most frequently used [26]. Current evidence supports DMSA or DMPS as first‐line agents due to their favorable safety profiles and efficacy, with BAL reserved for severe acute poisoning when parenteral therapy and intracellular mobilization are required [27].

It is important to note that while DMSA chelation is beneficial, its effectiveness may be limited in cases where renal failure is already significant. In such cases, extracorporeal therapies serve as important adjuncts. Hemodialysis helps in treating the renal failure caused by the ingestion of mercury and assists in correcting fluid and electrolyte imbalances [28]. Given mercury’s high protein binding and relatively low volume of distribution, plasmapheresis has emerged as a more effective modality for toxin removal [29, 30]. In one reported case, plasma exchange significantly reduced circulating mercury levels, whereas hemodialysis alone was ineffective; two sessions resulted in a measurable decline with a half‐life of approximately 23 days [31]. Plasmapheresis has also been successfully combined with chelation therapy in severe cases [32, 33].

Continuous venovenous hemodiafiltration (CVVHDF) offers an alternative in hemodynamically unstable patients or those requiring continuous renal support. When used in conjunction with chelation, CVVHDF has demonstrated clinically meaningful mercury clearance; in one severe case, 127 mg of mercury (12.7% of the ingested dose) was removed over 14 days, with complete clinical recovery [34]. However, compared to intermittent hemodialysis, continuous modalities may provide slower toxin clearance, which can limit their utility in acute poisoning [35, 36].

Collectively, these findings highlight that optimal management of acute mercuric chloride toxicity requires a multimodal approach, integrating early chelation with supportive care and, when indicated, targeted extracorporeal therapies. The choice of modality should be guided by the patient’s clinical status, timing of presentation, and extent of renal involvement, with the goal of minimizing systemic toxicity and facilitating renal recovery.

## 4. Conclusion

In conclusion, while acute mercuric chloride poisoning through ingestion is rare, it can lead to severe renal and gastrointestinal damage, as demonstrated in this case, where the patient developed ATN. Historically, patients with mercury poisoning, particularly those treated for syphilis, often succumbed to renal failure. However, in this case, timely treatment with DMSA chelation therapy effectively reduced systemic mercury levels, and hemodialysis played a crucial role in managing significant renal failure. The combination of these therapies, along with supportive care, facilitated the patient’s recovery and underscores the importance of early intervention in acute mercury toxicity. This case highlights the need for prompt, multidisciplinary management to optimize outcomes, particularly when renal function is compromised, and illustrates the critical role of hemodialysis in modern mercury poisoning treatment.

The consent for the case report has been obtained from the patient.

## Funding

This study was supported by HCA Healthcare.

## Conflicts of Interest

The authors declare no conflicts of interest.

## Data Availability

The data that support the findings of this study are available from the corresponding author upon reasonable request.

## References

[1] Broussard L. A. , Hammett-Stabler C. A. , Winecker R. E. , and Ropero-Miller J. D. , The Toxicology of Mercury, Laboratory Medicine. (2002) 33, no. 8, 614–625, 10.1309/5hy1-v3ne-2lfl-p9mt.

[2] Bernhoft R. A. , Mercury Toxicity and Treatment: a Review of the Literature, Journal of Environmental and Public Health. (2012) 2012, 460508–460510, 10.1155/2012/460508, 2-s2.0-84855572955. 22235210 PMC3253456

[3] Rakete S. , Asenbauer E. , Böhm S. et al., Mercury Poisoning of a 4-year-old Child by Indirect Contact with a mercury-containing Facial Cream: a Case Report, SAGE Open Medical Case Reports. (2021) 9, 10.1177/2050313x211025227. PMC8243084 34262770

[4] Rowens B. , Guerrero-Betancourt D. , Gottlieb C. A. , Boyes R. J. , and Eichenhorn M. S. , Respiratory Failure and Death Following Acute Inhalation of Mercury Vapor: a Clinical and Histologic Perspective, Chest. (1991) 99, no. 1, 185–190, 10.1378/chest.99.1.185, 2-s2.0-0026028707. 1984951

[5] Sarikaya S. , Karcioglu O. , Ay D. , Cetin A. , Aktas C. , and Serinken M. , Acute Mercury Poisoning: a Case Report, BMC Emergency Medicine. (2010) 10, no. 1, 10.1186/1471-227x-10-7, 2-s2.0-77951198793. PMC2848228 20302609

[6] Nayfeh A. , Kassim T. , Addasi N. , Alghoula F. , Holewinski C. , and Depew Z. , A Challenging Case of Acute Mercury Toxicity, Case Reports in Medicine. (2018) 2018, 1010678–4, 10.1155/2018/1010678, 2-s2.0-85043387283. 29559996 PMC5835301

[7] Parascandola J. , From Mercury to Miracle Drugs: Syphilis Therapy over the Centuries, Pharmacy in History. (2009) 51, no. 1, 14–23. 20027915

[8] Masur L. C. , A Review of the Use of Mercury in Historic and Current Ritualistic and Spiritual Practices, Alternative Medicine Review: A Journal of Clinical Therapeutics. (2011) 16, no. 4, 314–320. 22214251

[9] Rojas-Franco P. , Franco-Colín M. , Torres-Manzo A. P. et al., Endoplasmic Reticulum Stress Participates in the Pathophysiology of mercury-caused Acute Kidney Injury, Renal Failure. (2019) 41, no. 1, 1001–1010, 10.1080/0886022x.2019.1686019. 31736398 PMC6882499

[10] Krakowiak A. , Janasik B. , Sadowski Ł. , Szwabe K. , and Machała W. , Acute Mercuric Chloride Poisoning at a Potentially Lethal Dose Ended with Survival: Symptoms, Concentration in Cerebrospinal Fluid, Treatment, International Journal of Occupational Medicine & Environmental Health. (2023) 36, no. 5, 685–692, 10.13075/ijomeh.1896.02235. 37750691 PMC10702865

[11] Rafati-Rahimzadeh M. , Rafati-Rahimzadeh M. , Kazemi S. , and Moghadamnia A. A. , Current Approaches of the Management of Mercury Poisoning: Need of the Hour, Daru: Journal of Faculty of Pharmacy, Tehran University of Medical Sciences. (2014) 22, no. 1, 10.1186/2008-2231-22-46, 2-s2.0-84902543942. PMC4055906 24888360

[12] Parascandola J. , Syphilis: Its Early History and Treatment Until Penicillin and the Debate on Its Origins, Journal of Military and Veterans’ Health. (2019) 27, no. 2, 36–42.

[13] Ajsuvakova O. P. , Tinkov A. A. , Aschner M. et al., Sulfhydryl Groups as Targets of Mercury Toxicity, Coordination Chemistry Reviews. (2020) 417, 10.1016/j.ccr.2020.213343. PMC7470069 32905350

[14] Zalme R. C. , McDowell E. M. , Nagle R. B. , McNeil J. S. , Flamenbaum W. , and Trump B. F. , Studies on the Pathophysiology of Acute Renal Failure. Virchows Archiv, B Cell Pathology. (1976) 22, no. 1, 197–216, 10.1007/bf02889216, 2-s2.0-0017152915. 188227

[15] Li S. J. , Zhang S. H. , Chen H. P. et al., Mercury-Induced Membranous Nephropathy: Clinical and Pathological Features, Clinical Journal of the American Society of Nephrology. (2010) 5, no. 3, 439–444, 10.2215/cjn.07571009, 2-s2.0-77749282859. 20089494 PMC2827581

[16] Gao Z. , Wu N. , Du X. , Li H. , Mei X. , and Song Y. , Toxic Nephropathy Secondary to Chronic Mercury Poisoning: Clinical Characteristics and Outcomes, Kidney International Reports. (2022) 7, no. 6, 1189–1197, 10.1016/j.ekir.2022.03.009. 35694560 PMC9174032

[17] Bjørklund G. , Crisponi G. , Nurchi V. M. , Cappai R. , Buha Djordjevic A. , and Aaseth J. , A Review on Coordination Properties of thiol-containing Chelating Agents Towards Mercury, Cadmium, and Lead, Molecules. (2019) 24, no. 18, 10.3390/molecules24183247, 2-s2.0-85071777914. PMC6767255 31489907

[18] Kosnett M. J. , The Role of Chelation in the Treatment of Arsenic and Mercury Poisoning, Journal of Medical Toxicology: Official Journal of the American College of Medical Toxicology. (2013) 9, no. 4, 347–354, 10.1007/s13181-013-0344-5, 2-s2.0-84889238846. 24178900 PMC3846971

[19] Majdanik S. , Potocka-Banaś B. , Glowinski S. , and Luzny S. , Suicidal Intoxication with Mercury Chloride, Forensic Toxicology. (2023) 41, no. 2, 304–308, 10.1007/s11419-022-00653-7. 36564610 PMC10310567

[20] Bridges C. C. , Joshee L. , and Zalups R. K. , Multidrug Resistance Proteins and the Renal Elimination of Inorganic Mercury Mediated by 2,3-dimercaptopropane-1-sulfonic Acid and meso-2,3-dimercaptosuccinic Acid, Journal of Pharmacology and Experimental Therapeutics. (2008) 324, no. 1, 383–390, 10.1124/jpet.107.130708, 2-s2.0-37349054265. 17940195 PMC2409288

[21] Clarkson T. W. , Magos L. , Cox C. et al., Tests of Efficacy of Antidotes for Removal of Methylmercury in Human Poisoning During the Iraq Outbreak, Journal of Pharmacology and Experimental Therapeutics. (1981) 218, no. 1, 74–83, 10.1016/s0022-3565(25)32633-9. 7241391

[22] Aaseth J. , Skaug M. A. , Cao Y. , and Andersen O. , Chelation in Metal intoxication—Principles and Paradigms, Journal of Trace Elements in Medicine and Biology. (2015) 31, 260–266, 10.1016/j.jtemb.2014.10.001, 2-s2.0-84940163607. 25457281

[23] Andersen O. and Aaseth J. , A Review of Pitfalls and Progress in Chelation Treatment of Metal Poisonings, Journal of Trace Elements in Medicine and Biology. (2016) 38, 74–80, 10.1016/j.jtemb.2016.03.013, 2-s2.0-84963625856. 27150911

[24] Cao Y. , Skaug M. A. , Andersen O. , and Aaseth J. , Chelation Therapy in Intoxications with Mercury, Lead and Copper, Journal of Trace Elements in Medicine and Biology. (2015) 31, 188–192, 10.1016/j.jtemb.2014.04.010, 2-s2.0-84929656616. 24894443

[25] Rush T. , Hjelmhaug J. , and Lobner D. , Effects of Chelators on Mercury, Iron, and Lead Neurotoxicity in Cortical Culture, Neurotoxicology. (2009) 30, no. 1, 47–51, 10.1016/j.neuro.2008.10.009, 2-s2.0-58349120238. 19027035

[26] Shi Y. , Wang M. , Ni H. et al., Clinical Characteristics, Management, and Outcomes of Diseases Caused by Mercury Overexposure: a Systematic Review of Case Reports and Case Series, Frontiers in Public Health. (2026) 14, 10.3389/fpubh.2026.1750332. PMC12907422 41705054

[27] Aaseth J. , Skaug M. A. , Cao Y. , and Andersen O. , Chelation in Metal intoxication—Principles and Paradigms, Journal of Trace Elements in Medicine and Biology. (2015) 31, 260–266, 10.1016/j.jtemb.2014.10.001, 2-s2.0-84940163607. 25457281

[28] Ye B. J. , Kim B. G. , Jeon M. J. et al., Evaluation of Mercury Exposure Level, Clinical Diagnosis, and Treatment for Mercury Intoxication, Annals of Occupational and Environmental Medicine. (2016) 28, no. 1, 10.1186/s40557-015-0086-8, 2-s2.0-84996477507. PMC4724159 26807265

[29] Nenov V. D. , Marinov P. , Sabeva J. , and Nenov D. S. , Current Applications of Plasmapheresis in Clinical Toxicology, Nephrology Dialysis Transplantation. (2003) 18, no. Suppl 5, v56–v58, 10.1093/ndt/gfg1049. 12817073

[30] Schutt R. C. , Ronco C. , and Rosner M. H. , The Role of Therapeutic Plasma Exchange in Poisonings and Intoxications, Seminars in Dialysis. (2012) 25, no. 2, 201–206, 10.1111/j.1525-139x.2011.01033.x, 2-s2.0-84858707274. 22353434

[31] Yoshida M. , Satoh H. , Igarashi M. , Akashi K. , Yamamura Y. , and Yoshida K. , Acute Mercury Poisoning by Intentional Ingestion of Mercuric Chloride, Tohoku Journal of Experimental Medicine. (1997) 182, no. 4, 347–352, 10.1620/tjem.182.347, 2-s2.0-0031442238. 9352627

[32] Lu Q. , Liu Z. , and Chen X. , Mercury Poisoning Through Intravenous Administration: Two Case Reports with Literature Review, Medicine. (2017) 96, no. 46, 10.1097/md.0000000000008643, 2-s2.0-85037713687. PMC5704834 29145289

[33] Zou M. , Li J. , Yao H. et al., Toxic Encephalopathy due to Mercury: a Rare Case Report and Literature Review, Medicine. (2025) 104, no. 37, 10.1097/md.0000000000044037. PMC12440396 40958342

[34] Dargan P. I. , Giles L. J. , Wallace C. I. et al., Severe Mercuric Sulphate Poisoning Treated with 2,3-dimercaptopropane-1-sulphonate and Haemodiafiltration: a Case Report, Critical Care. (2003) 7, no. 3, R1–R6, 10.1186/cc1887. 12793883 PMC270669

[35] Goodman J. W. and Goldfarb D. S. , The Role of Continuous Renal Replacement Therapy in the Treatment of Poisoning, Seminars in Dialysis. (2006) 19, no. 5, 402–407, 10.1111/j.1525-139x.2006.00194.x, 2-s2.0-33748691152. 16970740

[36] Cabrera V. J. and Shirali A. C. , We Use Continuous Renal Replacement Therapy for Overdoses and Intoxications, Seminars in Dialysis. (2016) 29, no. 4, 275–277, 10.1111/sdi.12505, 2-s2.0-84977580236. 27126739

